# Rationale and design: telephone-delivered behavioral skills interventions for Blacks with type 2 diabetes

**DOI:** 10.1186/1745-6215-11-35

**Published:** 2010-03-29

**Authors:** Leonard E Egede, Joni L Strom, Valerie L Durkalski, Patrick D Mauldin, William P Moran

**Affiliations:** 1 Center for Disease Prevention and Health Interventions for Diverse Populations, Ralph H. Johnson VAMC, Charleston, SC, USA; 2 Division of General Internal Medicine & Geriatrics, Medical University of South Carolina, Charleston, South Carolina, USA; 3 Center for Health Disparities Research, Medical University of South Carolina, Charleston, South Carolina, USA; 4 Division of Biostatistics and Epidemiology, Medical University of South Carolina, Charleston, South Carolina, USA; 5 Department of Clinical Pharmacy and Outcome Sciences, Medical University of South Carolina, Charleston, South Carolina, USA

## Abstract

**Background:**

African Americans with Type 2 diabetes (T2DM) have higher prevalence of diabetes, poorer metabolic control, and greater risk for complications and death compared to American Whites. Poor outcomes in African Americans with T2DM can be attributed to patient, provider, and health systems level factors. Provider and health system factors account for <10% of variance in major diabetes outcomes including hemoglobin A1c (HbA1c), lipid control, and resource use. Key differences appear to be at the patient level. Of the patient level factors, consistent differences between African Americans and American Whites with T2DM have been found in diabetes knowledge, self-management skills, empowerment, and perceived control. A variety of interventions to improve diabetes self-management have been tested including: 1) knowledge interventions; 2) lifestyle interventions; 3) skills training interventions; and 4) patient activation and empowerment interventions. Most of these interventions have been tested individually, but rarely have they been tested in combination, especially among African Americans who have the greatest burden of diabetes related complications. This study provides a unique opportunity to address this gap in the literature.

**Methods/Design:**

We describe an ongoing four-year randomized clinical trial, using a 2 × 2 factorial design, which will test the efficacy of separate and combined telephone-delivered, diabetes knowledge/information and motivation/behavioral skills training interventions in high risk African Americans with poorly controlled T2DM (HbA1c ≥ 9%). Two-hundred thirty-two (232) male and female African-American participants, 18 years of age or older and with an HbA1c ≥ 9%, will be randomized into one of four groups for 12-weeks of phone interventions: (1) an education group, (2) a motivation/skills group, (3) a combined group or (4) a usual care/general health education group. Participants will be followed for 12-months to ascertain the effect of the interventions on glycemic control. Our primary hypothesis is that among African Americans with poorly controlled T2DM, patients randomized to the combined diabetes knowledge/information and motivation/behavioral skills training intervention will have significantly greater reduction in HbA1c at 12 months of follow-up compared to the usual care/general health education group.

**Discussion:**

Results from this study will provide important insight into how best to deliver diabetes education and skills training in ethnic minorities and whether combined knowledge/information and motivation/behavioral skills training is superior to the usual method of delivering diabetes education for African Americans with poorly controlled T2DM.

**Trial registration:**

National Institutes of Health Clinical Trials Registry (ClinicalTrials.gov identifier# NCT00929838).

## Background

Diabetes affects approximately 23.6 million people or 7.8% of the United States population [1]. Diabetes is associated with significant morbidity, mortality, increased health care utilization, and increased health care costs [1]. Diabetes is the leading cause of cardiovascular disease (CVD), strokes, blindness, and lower limb amputations [2]. It was the seventh leading cause of death listed on U.S. death certificates in 2006, and individuals with diabetes have a two-fold increased risk of death compared to individuals without diabetes of similar age and sex [1]. Type 2 diabetes accounts for 90-95% of all cases of diabetes in the United States [1].

African Americans are almost twice as likely to have diabetes as white Americans of similar age [1]. Approximately 14.7% of African Americans over age 20 years have diabetes compared to 9.8% of non-Hispanic American Whites [1]. The prevalence of diabetes in African Americans has increased dramatically in recent times [1]. Type 2 diabetes is the most prevalent type of diabetes in African Americans accounting for 90 to 95% of all cases [1]. African Americans have higher incidence of and greater disability from diabetes complications than white Americans [1]. African Americans with diabetes are four-times more likely to develop ESRD than their American White counterparts with diabetes [1]. Diabetic retinopathy is 40-50% more frequent in African Americans than in white Americans [1]. Also, African Americans with diabetes are much more likely to undergo a lower-extremity amputation than white or Hispanic Americans with diabetes [1]. Death rates for people with diabetes are 20-40 percent higher in African Americans compared with white Americans [1]. Thus, evidence indicates that diabetes is a significant public health problem; and African Americans appear disproportionately burdened with the complications and disability that result from poorly treated diabetes.

Data from recent studies have shown that Blacks have poorer glycemic and blood pressure control than American Whites [3-5]. In a nationally representative sample, [4] after controlling for relevant covariates, African Americans with diabetes were more likely to have poor glycemic control and poorly controlled blood pressure. In a more recent study [5], national trends in processes of care and intermediate outcomes for diabetes between 1988-1994 and 1999-2002 were compared using national data. The study showed that although diabetes processes of care and intermediate outcomes have improved nationally, 1 in 3 persons still have poor blood pressure control, and 1 in 5 persons still have poor glycemic control. Ethnic minority patients (including African Americans) had poorer improvements in glycemic and blood pressure control over the study period.

Several reasons contribute to poor diabetes outcomes in African Americans. These include factors at the patient, provider, and health systems levels. Patient level factors include poor diabetes-specific knowledge, negative belief and attitudes about diabetes, lack of self-management skills, and non-adherence to lifestyle behaviors [6-8]. Other factors include mismatch of patient and physician expectations, differential socioeconomic levels that impede physician-patient communication, and distrust of physicians that may decrease adherence [9,10]. Language barriers and low literacy rates also impede physician-patient communication [10,11]. Other important patient level factors include lack of a locus of control [12] and fatalism [13]. Provider level factors include negative beliefs and attitudes about diabetes [14], perceived complexity and difficulty of treating diabetes [15,16], lack of adequate time and resources for diabetes treatment [16,17] and clinical inertia [18-20]. Health systems factors include accessibility, availability and convenience of appointments, organization of care, availability of interpreters, health insurance coverage, reimbursement levels, and formulary restrictions [21].

While provider and health system level factors are important for improved glycemic control, they explain <10% of variance in diabetes outcomes including glycemic control, lipid control, and resource use/cost [22,23]. Most of the variation in diabetes outcomes is due to patient level factors. Of the patient level factors, consistent differences between African Americans and American Whites with T2DM have been found in diabetes knowledge, self-management skills, empowerment, and perceived control [6-8,12,13,21].

This paper describes the rationale, study aims and objectives, and research design and methods of an ongoing four-year, randomized clinical trial testing the efficacy of separate and combined telephone-delivered, diabetes knowledge/information and motivation/behavioral skills training intervention in high risk African Americans with poorly controlled T2DM (HbA1c ≥ 9%). The long-term goal of the project is to achieve improvement in diabetes-related outcomes in this patient population.

### Rationale

Despite the compelling evidence of the effectiveness of diabetes self-management interventions, several limitations hinder their generalizability to high risk African Americans with T2DM. *First*, few studies recruited sufficient number of African Americans with T2DM to establish the effectiveness of these interventions in this patient population. *Second*, there are no randomized clinical trials that have compared diabetes knowledge/information, motivation/behavioral skills training, or the combination of both interventions in African Americans with T2DM. Most prior studies have tested these interventions individually. Rarely have they been tested in combination or compared against one another in a randomized clinical trial. Given the high burden of disease and poorer outcomes in African Americans, it is critical to identify effective interventions for this population. *Third*, internal validity was frequently threatened by lack of blinding of the assessors, contamination of the control group, unintended co-interventions, and deficits in the instruments used to measure diabetes knowledge, self-care, and dietary habits. These methodological limitations were noted in the systematic review of effectiveness of diabetes self-management intervention in T2DM by Norris and colleagues [24]. *Fourth*, earlier interventions were of limited dose and intensity, patients had to address multiple behaviors at the same time, and patients were typically not given the option to select the behaviors they wanted to address [24]. *Finally*, the behavioral theories on which interventions were based were not adequately described or documented in most studies [24]. Accordingly, in this study, we evaluate the efficacy of a combined telephone-delivered, diabetes knowledge/information and motivation/behavioral skills training intervention targeting physical activity, diet, medication adherence, and self blood glucose monitoring in improving HbA1c levels in high risk African Americans with poorly controlled T2DM. In addition, we minimize the methodological limitations of prior patient-level interventions by eliminating threats to internal validity, basing the interventions on established behavioral theory, and delivering adequate dose and intensity of the interventions based on findings from our preliminary studies.

### Study Aims & Objectives

The primary objective of this study is to test the separate and combined efficacy of a telephone-delivered diabetes knowledge/information and motivation/behavioral skills training intervention in improving HbA1c levels in African Americans with T2DM using a 2 × 2 factorial design.

## Methods/Design

The study is a 4 group randomized controlled trial with randomization of individual participants, blinded outcomes assessments at baseline, 3-months, 6-months, and 12-months, and concurrent economic evaluation.

### Location and Setting

The study sites for this study include the general internal medicine, endocrine, family medicine, and community care clinics affiliated with the Medical University of South Carolina in Charleston, SC and the primary care and endocrine clinics of the Ralph H. Johnson VA Medical Center in Charleston, SC.

### Ethics and Trial Registration

The study is funded by grant R01DK081121-01A1 from the National Institute of Diabetes and Digestive and Kidney Diseases (NIDDK). The trial is approved by the Institutional Review Board (IRB) of the Medical University of South Carolina (HR#18334). The trial is registered on the United States National Institutes of Health Clinical Trials Registry (ClinicalTrials.gov identifier# NCT00929838), available online at: http://clinicaltrials.gov/ct2/show/NCT00929838.

### Trial Population and Recruitment

A total of 232 African Americans with T2DM will be randomized to one of four groups: 1) telephone-delivered diabetes knowledge/information; 2) telephone-delivered motivation/behavioral skills training; 3) combined telephone-delivered diabetes knowledge/information and motivation/behavioral skills training; and 4) usual care.

We use two complementary approaches to identify eligible study participants. The first method consists of systematic identification of African American patients with T2DM. After obtaining approval for a partial waiver of HIPAA from our local institutional review board (IRB) and the Research and Development committee of the Ralph H. Johnson Medical Center, we use clinic-billing records over the previous 12-month period to identify African American participants with ICD-9 codes consistent with a diagnosis of type 2 diabetes. The physicians of eligible patients are notified of their patients' potential eligibility and asked permission to enroll their patients in this study. After consent is obtained from the physicians, letters of invitation on clinic letterhead signed by the patient's physician are mailed to patients from the study clinics. The letter provides information about the study, explains the study requirements, and clarifies that only participants who meet certain criteria will be eligible to participate in the study. The letter includes an addressed and stamped post-card that participants can mail back to indicate interest or lack of interest in participating in the study. In addition, the letter provides a telephone number that interested participants can call to receive detailed information about the study. In the letter, participants are also informed that they will receive a follow-up call in two weeks unless they mail back the post card or call to decline being contacted. Participants who mail back the post card and express interest or call the provided telephone number receive detailed information about the study. Participants who agree to participate are asked to provide written consent and are scheduled for the initial screening assessment.

The second method consists of referrals from physicians, other clinic staff such as nurses, or patients themselves in response to recruitment flyers for the study. The PI shares the goals of the study and inclusion/exclusion criteria with physicians and clinic staff during clinic administrative meetings. Physicians and clinic staff are asked to refer appropriate participants to the study research assistants. In addition, IRB approved recruitment flyers are posted in prominent locations in the study clinics.

Regardless of recruitment pathway, research staffs obtain written informed consent, complete screening for eligibility, and assure that participants meet criteria for inclusion and participation in the study. The procedure and risks are explained to the patients and the consent form signed as per standard clinical practice. Participants who meet eligibility criteria then complete the remainder of the assessment battery (see Tables 1, 2 and 3).

**Table 1:** Data Collection Schedule

Questionnaires/Measurements	Baseline Visit	3-month Visit	6-month visit	12-month visit
**Primary Outcome Measure**				

HbA1c	X	X	X	X

Fasting lipid profile	X	X	X	X

				

**Secondary Outcome Measures**				

Physical Activity	X	X	X	X

Diet	X	X	X	X

Medication Adherence (eCaps)	X	X	X	X

Self-Monitoring of Blood Glucose	X	X	X	

Blood Pressure	X	X	X	X

Resource Use/Cost	X	X	X	X

Body Composition (BOD POD)	X			X

Other Laboratory measurements	X			X

Anthropometric measurements	X	X	X	X

				

**Process Measures**				

Diabetes Knowledge Questionnaire	X	X	X	X

Diabetes Empowerment Scale	X	X	X	X

Summary of Diabetes Self-Care Activities Scale	X	X	X	X

Treatment Credibility		X	X	

Perceived Diabetes Self Efficacy Scale	X	X	X	X

				

**Self-report Measures**				

Patient Demographics	X			

Quality of life (SF-12)	X			X

Social support	X			X

Health Literacy	X			X

Depression (PHQ-9)	X	X	X	X

Medical Comorbidity (Charlson Index)	X			X

Morisky Medication Adherence Scale	X	X	X	X

Diabetes Fatalism Scale	X	X	X	X

Service Delivery Perceptions		X	X	

Assessment of eCaps Use		X	X	X

**Table 2:** Data Collection Measures

Outcome	Test	Measurement
**Primary Outcome Measures**	HbA1c/Fasting Lipid Profile	Blood specimens will be obtained at baseline, 3-, 6- and 12-months visits.

**Secondary Outcome Measures**	Laboratory Measurements	Blood specimens will be obtained at baseline and 12-months visits.

	Physical Activity	The seven-day physical activity recall (PAR) will be used to measure of physical activity [29,30].

	Diet	Dietary intake will be assessed using the Block 1998 Food Frequency Questionnaire [31-36].

	Medication Adherence	The eCAP^® ^electronic compliance monitor (Information Mediary Corporation, Ottawa, Canada) will serve as the primary measure of medication adherence for this study.

	Self-monitoring of Blood Glucose	Glucometer downloads will be used to assess self-monitoring adherence.

	Blood pressure measurements	Blood pressure readings will be obtained at baseline, 3-, 6- and 12-months by a trained RA using automated BP monitors (OMRON IntelliSense™ HEM-907XL) with the patient seated comfortably for 5 minutes prior to the measurements.

	Resource Utilization & Cost	The perspective of cost will be that of the payer. Previously validated questions on resource utilization will be administered as part of the baseline, 3-, 6-, and 12-month assessments.

	Body Composition	The BOD POD *Gold Standard *(Life Measurement, Inc., Concord, CA) will serve as the primary measure of body composition for this study.

	Anthropometrics	Measurements of body fat including height, weight, BMI, waist circumference, waist-to-hip ratio, and skinfold thickness will be obtained at baseline, 3-, 6-, and 12-month visits.

**Table 3:** Data Collection Instruments

Measure	Data Collected	Method
**Process Measures**	Information	This will be measured by the 24-item Diabetes Knowledge Questionnaire (DKQ) [37].

	Motivation	This will be measured by the 8-item Diabetes Empowerment Scale-Short Form (DES-SF) [38].

	Self-Efficacy	This will be measured by the perceived diabetes self-management scale (PDSMS) [39].

	Behavioral Skills	This will be assessed with the Summary of Diabetes Self-Care Activities (SDSCA) scale [40].

	Treatment Credibility	To assess for differences in outcome expectancy, a modified treatment credibility scale developed by Borkovec and Nau (1972) will be used [41].

**Self-report measures**	Demographics	Previously validated items from the 2002 National Health Interview Survey [42] will be used to capture age, gender, race/ethnicity, marital status, household income, and health insurance.

	Quality of Life	Quality of life will be measured by the SF-12 [43], which is a valid and reliable instrument to measure functional status.

	Social support	The Medical Outcomes Study (MOS) Social Support Survey [44] will be used to measure social support.

	Health Literacy	The abbreviated version of the Test of Functional Health Literacy in Adults (S-TOFHLA) [45] is designed to rapidly screen patients for potential health literacy problems.

	Depression	The PHQ-9 is a brief questionnaire that scores each of the 9 DSM-IV criteria for depression [46].

	Medical Comorbidity	The patient's history of medical comorbidity will be documented using a standardized and validated questionnaire(ref#200) [42].

	Self-Reported Medication Adherence	This will be measured with the new 8-item self-report Morisky Medication Adherence Scale (MMAS) [47].

	Diabetes Fatalism	This will be measured with the 12-item Diabetes Fatalism Scales (DFS) [48].

	Service Delivery Perceptions	This will be assessed with 5 items that have been previously validated in mental health studies. The items were slightly modified to be relevant to diabetes.

	Assessment of ECAPS Use	A brief 8-item scale will be used to assess the ease of use of the electronic medication adherence pill bottle.

### Randomization

All participants are randomly assigned to one of the four study arms (n = 58 per arm). Randomization takes place in waves. Approximately 58 participants are randomized every 6 months. The randomization sequence is generated by a web-based computer generated randomization scheme. After determining eligibility, enrolled patients are assigned to treatment groups by the health educators. The health educators log into the web-based program and determine which of the 4 groups they are randomized to. This information is confidential and not shared with the study investigators in accordance with the CONSORT guidelines [25]. Once a randomization assignment is provided, the patient is entered into the study and is included in the intention to treat analysis.

### Intervention and Control Groups

There are three active treatment groups (telephone-delivered diabetes knowledge/information, telephone-delivered motivation/behavioral skills training, combined telephone-delivered diabetes knowledge/information and telephone-delivered motivation/behavioral skills training) and a usual care/health education group.

The patient-level intervention is based on the information-motivation-behavioral skills model and provides information, motivation, and behavioral skills training. Study assessments are performed by a blinded research assistant at baseline, 3, 6, and 12 months of follow-up. The primary outcome is HbA1c level at 12 months of follow-up.

### Contents of Individual Treatment Sessions by Intervention Group

#### Session 1

After enrollment, randomization, and completing the baseline assessment, each participant comes in for a face-to-face meeting with the study health educators regardless of the group randomized to. During this visit, the health educators goes over the study in detail, obtains patient contact information, primary and alternate telephone numbers, and establishes guidelines for follow-up calls. In addition, participants receive information specific to their group as described below:

##### a. Education group

Participants randomized to this group receive a culturally tailored education booklet that was developed as part of REACH 2010 titled "Your Guide to Sugar Diabetes". This 48 page document is written in lay language (6^th ^grade education) for African Americans with diabetes. Participants are encouraged to review the materials and discuss questions during subsequent diabetes education telephone calls.

The diabetes knowledge/information intervention for the participants randomized to this group consists of weekly telephone-delivered diabetes knowledge/information lasting 30 minutes for 12 weeks based on American Diabetes Association (ADA) guidelines. After initially meeting for session 1, all subsequent sessions (2-13) are delivered via telephone by the health educators. Participants randomized to this group participate in discussions on diabetes-related topics, including an overview of diabetes, self-blood glucose monitoring, medication adherence, the basics of eating, meal planning, carbohydrate counting, exercise, blood pressure, cholesterol, foot and skin care, and controlling stress levels. At the completion of session 13, the health educators conduct a review session, highlighting and summarizing the major points from the previous sessions.

##### b. Behavioral skills training intervention group

Participants randomized to this group are trained on how to use the 5 patient activation questions during clinic visits. They are given the patient empowerment package ("Diabetes Care Kit") and trained on how to use the materials in the diabetes care package. In addition, the health educator goes over the principles of behavioral skills training, asks the patient to choose the first target behavior, and assists the subject in developing an action plan.

The motivation/behavioral skills intervention consists of patient activation (list of 5 questions to ask their provider at every visit and training on how to ask the questions), patient empowerment (diabetes responsibility contracts and flow charts for patients to record lab results/medications and training on how to use the flow charts), and behavioral skills training delivered via telephone lasting 30 minutes every week for 12 weeks. Four topics-self-blood sugar monitoring, medication adherence, diet, and physical activity-are stressed in these sessions (2-13). Participants randomized to this group have the option to select the order in which these topics are discussed. During these sessions, the importance of the selected behavior in diabetes management, as well as the participants' motivation towards changing and/or maintaining this behavior are assessed by the health educator. The health educator asks about the participants' current status and practices of the behavior, in addition to any barriers and limitations they face in controlling that particular behavior. At the end of each session, the participants have to set a goal to practice over the next week. During the next session, the health educator checks the participant's progress of that goal and his/her confidence in maintaining that behavior.

##### c. Combined intervention group

Participants randomized to this group receive the culturally tailored education booklet that was developed as part of REACH 2010 titled "Your Guide to Sugar Diabetes". They are trained on how to use the 5 patient activation questions during clinic visits. They are also given the patient empowerment package and trained on how to use the materials in the diabetes care package. In addition, the health educator goes over the principles of behavioral skills training, asks the subject to chose the first target behavior, and assists the subject in developing an action plan.

The combined intervention group receives weekly telephone-delivered diabetes knowledge/information, patient activation, patient empowerment, and behavioral skills training delivered via telephone. Telephone sessions (2-13) for the combined intervention group are delivered weekly for 12 weeks and last for 30 minutes. Participants randomized to this group participate in discussions on diabetes-related topics and receive motivation/skills training in self-blood sugar monitoring, medication adherence, diet, and physical activity, as outlined in the education group and the behavioral skills training group above. During the final week of discussion, a review of the major points of each session covered previously will be conducted.

##### d. Usual care group

Participants randomized to this group receive general health information booklets. The men receive a 23-page booklet titled "What I Need to Know about Prostate Problems", while the women receive a 19-page booklet titled "What I Need to Know about Urinary Tract Infections". Both booklets are written in lay language. Participants are encouraged to review the materials and discuss questions during subsequent education telephone calls.

The usual care group receives weekly telephone-delivered general health education (sessions 2-13) lasting 30 minutes every week for 12 weeks to control for attention. Participants randomized to this group discuss general health education topics which include managing back pain, sleep problems, stroke and transient ischemic attacks (TIAs), vitamins, health care insurance, hepatitis viruses, influenza and pneumonia, dyspepsia, colon cancer, migraine headaches, sore throat, and esophageal reflux (GERD).

At the completion of session 13, regardless of the assigned group, all participants are congratulated on their success and scheduled for a 3-month follow-up appointment.

Several intervention materials used in the study are copyrighted and cannot be made publicly available without the consent of the copyright owners. However, manuals developed as part of this study will be shared in a timely fashion with researchers in both the private and public sector for a nominal charge and with minimal restriction in accordance with United States National Institute of Health and our institutional guidelines.

### Study Instruments and Data Collection Schedule

**Figure 1:**
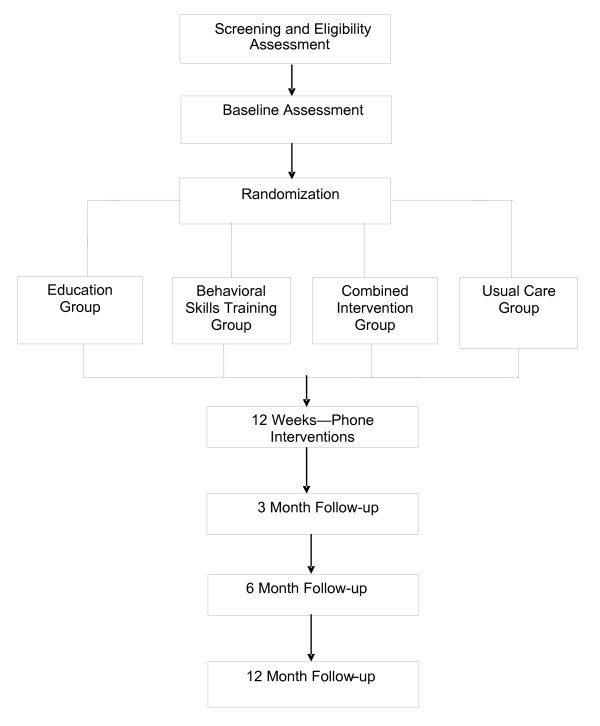
**Design and Study Flow**.

See Figure 1 and Tables 1, 2 and 3, for the study design and study flow, data collection schedule, data collection measures, and data collection instruments, respectively.

### Intervention Delivery, Treatment Integrity, and Treatment Adherence

Two full-time masters-level trained health educators (HE) deliver the interventions. Each HE provides care to the participants in all of the four study groups.

#### Training and supervision of interventionists (health educators)

Training in the basic elements of behavioral skills training and ongoing supervision and oversight of the coaching sessions is provided by the study consultant (psychologist). Training consists of two full days of information and role-playing in behavioral skills training in year 01 and then two day booster sessions in years 2-4 to minimize drift in counseling skills. The Principal Investigator (PI) trains the health educators on content and conduct of diabetes and general health education sessions. The initial training involves role plays, and basic skills in running of telephone sessions. Additional training on the behavioral framework on which the intervention is based is also provided.

#### Treatment fidelity checks

In order to ensure that treatments are competently administered in accordance with the study protocol, all sessions are audiotaped, and 20% of these are randomly selected and rated for competence and adherence by co-investigators. This allows assessment for group differences on non-specific factors, such as interventionist empathy and rapport. To evaluate interventionist adherence, rating forms were developed based upon the treatment protocol to determine if the health educators appropriately covered the content of each session (i.e., demonstrated the particular behavior described in each item). To evaluate competence, rating forms were developed to assess how well the health educators accomplish a range of relevant tasks for each session (i.e., how well they carried out the particular behaviors described in each item). These rating forms use Likert scale response formats, and are modeled after the treatment fidelity forms used in other studies by the PI and members of the team. Review and rating of a random sample of audiotapes is used to assess for fidelity to treatment.

#### Patient compliance with treatment protocol

This is a very important aspect of the study given the intensity of the intervention protocol. We adopted the following strategies to ensure an optimal level of compliance: (1) at enrollment the health educators stress the importance of attending the sessions; (2) the health educators place reminder telephone calls to participants on the day or evening prior to each session; (3) the health educators request the names and telephone numbers of three of the participant's friends and/or relatives who know how to reach the participant in the event the team is unable to reach them; (4) the research staff are flexible in accommodating participants' schedules, so the telephone sessions can be conducted at times convenient for the participant; (5) participants receive a welcome package at enrollment and cards on birthdays or significant life events. In addition, participants are sent newsletters with useful information and testimonials from other study participants every 6 months to improve retention. Finally, participants are reimbursed $25 for the baseline, 3-, 6-, and 12-month follow-up visits for a total of $100 over the study duration.

#### Treatment enactment

The 4 behaviors that are the target of this intervention are physical activity, diet, medication adherence, and glucose self-monitoring. Changes in these behaviors are being assessed as measures of treatment enactment.

### Primary Outcome Measure

The primary outcome is HbA1c level at 12 months of follow-up.

### Sample Size Determination and Power Analysis

The sample size calculations are based on testing the three hypotheses in the 2 × 2 factorial design: 1) the overall effect of diabetes knowledge/information versus no diabetes knowledge/information, 2) the overall effect of motivation/behavioral skills versus no motivation/behavioral skills, and 3) the effect of the combined interventions. The primary endpoint for testing these hypotheses is the 12 month HbA1c value. The clinically relevant difference in HbA1c at 12 months is 1-percentage point and is based upon the findings of a previous RCT of telephone-delivered diabetes intervention [26] and our pilot study. In the study by Piette and colleagues [26], they found that after 12 months, the telephone intervention group lowered HbA1c by 1.1% when the baseline HbA1c was ≥ 9%. In our pilot study, we found that after 6 months, the telephone intervention group lowered HbA1c by 0.95% (sd 1.2), while the control group increased HbA1c by 0.3% (sd 1.5) (p = 0.06).

Sample size estimation is based on a 2 × 2 factorial design and takes into account the potential interaction between the diabetes knowledge/information and motivation/behavioral skills intervention. Although the common advantage of a 2 × 2 factorial design is the conservation of sample size in the absence of interaction, the alternative advantage is a gain in information on the interaction between two interventions. A total of 42 participants per arm is required to achieve 85% power (type I error rate of 0.05) to detect a clinically relevant difference of 1 percentage point in the 12-month HbA1c (assuming a common standard deviation of 1.5). Because testing for an interaction can greatly increase the sample size and since interaction is not our specific interest, we chose to ensure that we have adequate power to detect main effects in the presence of an interaction. Thus the total number of participants required for randomization is 168 participants (42 per treatment arm). The total sample size is inflated by a factor of 1.38 to account for an anticipated 15% attrition rate in an intent-to-treat analysis [27]. Thus a total of 232 participants will be randomized into this study. In the absence of interaction, the test comparing telephone-delivered diabetes knowledge/information versus usual care has greater than 95% power at a significance level of 0.025 to detect a difference of 1 percentage point. The test comparing telephone-delivered motivation/behavioral skills training versus usual care has the same power to detect a similar difference.

### Data Analysis

#### Primary Hypotheses

Among African Americans with poorly controlled T2DM, patients randomized to the telephone-delivered diabetes knowledge/information intervention, the telephone-delivered motivation/behavioral skills training intervention or the combined intervention will have significantly greater reduction in HbA1c at 12 months of follow-up compared to usual care.

The primary framework for analysis is analysis of covariance. Twelve-month HbA1c measurements (dependent variable) will be compared between treatment arms using a model-based approach with telephone-delivered diabetes knowledge/information, telephone-delivered motivation/behavioral skills training and the interaction of the two factors in the model along with baseline HbA1c as a covariate. A global test approach will be implemented in order to preserve the type I error rate [28]. The first test to be conducted is an overall test of differences among the four treatment arms using a type I error rate of 0.05. If the test on the null hypothesis of all four arms being equal is not significant then we will conclude that usual care is the treatment of choice with no further testing. If the test is significant then we will proceed with a test for interaction at the 0.10 significance level. If the interaction is not significant and either of the main treatment effects is significant, we will examine the effect on HbA1c in those participants assigned to the respective treatment versus those not assigned. If the interaction test is significant and favorable, usual care versus telephone-delivered diabetes knowledge/information and usual care versus telephone-delivered motivation/behavioral skills training will be tested. If both are significant, the combination of the two will be considered the best treatment. If only one is significant, then a test of the one intervention versus the combination will be conducted, and if neither are significant than the combination will be compared against usual care. There is no clinical/scientific reason to expect an unfavorable interaction; however, if one is detected the comparison of treatments to usual care will be conducted to determine which treatment is superior. All primary comparison tests will be conducted at the 0.05 significance level.

A mixed model repeated measures analysis of covariance will be conducted as a secondary analysis to assess the difference in HbA1c levels over time. The model will include telephone-delivered diabetes knowledge/information and telephone-delivered motivation/behavioral skills training and the interaction of the two as factors in the model along with baseline HbA1c as a covariate. A similar analysis plan using a global test approach as describe above will be implemented. If the interaction is not significant, it will be dropped from the model. Covariance structures will be assessed during the model-fitting process.

## Discussion

The study was funded in August 2008. Study recruitment began in May 2009, with all follow-up assessments associated with the study expected to be completed by June 2012. As of March 24 2010, 176 have been scheduled, 104 enrolled, and 95 randomized. Of the number randomized, 61 have completed the 12-week phone interventions, and 52 have completed the 3-month follow-up assessment and 33 have completed the 6-months follow-up assessment.

The proposed study provides a unique opportunity to address existing gaps in the literature by testing a combined telephone-delivered, diabetes knowledge/information and motivation/behavioral skills training intervention in African Americans with poorly controlled T2DM. The combined diabetes knowledge/information and motivation/behavioral skills intervention focuses on four key behaviors (physical activity, diet, medication adherence, and self-monitoring of blood glucose); maintain adequate dose and intensity of the interventions by delivering the interventions weekly for 12 weeks; and allow patients to choose one behavior to address every 3 weeks. The findings of this study, if successful, will lead to the implementation of this feasible, evidence-based intervention for high risk minority patients with T2DM. The study will provide new information on how to improve quality of care for diabetes in ethnic minorities and reduce the disproportionate burden of diabetes complications and deaths in ethnic minority groups with T2DM.

## Abbreviations

ADA: American Diabetes Association; ANCOVA: Analysis of Covariance; CBOC: Community Based Outpatient Clinic; CONSORT: Consolidated Standards of Reporting Trials; CVD: Cardiovascular Disease; ESRD: End Stage Renal Disease; GERD: Gastroesophageal Reflux; HbA1c: Hemoglobin A1c; HE: Health Educator; HIPAA: Health Insurance Portability and Accountability Act; ICD-9: The International Classification of Diseases, 9^th ^revision; IRB: Institutional Review Board; NIDDK: National Institute of Diabetes and Digestive and Kidney Diseases; PI: Principal Investigator; RCT: Randomized Control Trial; REACH 2010: Racial and Ethnic Approaches to Community Health 2010; T2DM: Type 2 Diabetes Mellitus; TIA: Transient Ischemic Attacks

## Competing interests

The authors declare that they have no competing interests.

## Authors' contributions

LEE conceived of the study; LEE obtained funding for the study; LEE, VLD, PDM, and WPM participated in the design and coordination of the study and reviewed the manuscript. JLS and LEE drafted and finalized the manuscript. All authors read and approved the final manuscript.
